# Non-linear associations and threshold effects of oxidative balance score and composite dietary antioxidant index on frailty risk in patients with cardiovascular-kidney-metabolic syndrome

**DOI:** 10.3389/fnut.2025.1673736

**Published:** 2025-11-03

**Authors:** Zheng Wang, Lei Fang, Long Wang, Jing Zhang

**Affiliations:** ^1^Department of Cardiology, The Second People’s Hospital of Hefei, Hefei Hospital Affiliated to Anhui Medical University, Hefei, Anhui, China; ^2^Department of Geriatrics Center, Tongling People ‘s Hospital, Tongling, Anhui, China

**Keywords:** oxidative balance score, comprehensive dietary antioxidant index, cardiovascular-kidney-metabolic syndrome, frailty, non-linear association, additive effect, NHANES

## Abstract

**Objective:**

The aim of this study was to investigate the independent and combined effects of oxidative balance score (OBS) and composite dietary antioxidant index (CDAI) on the risk of frailty in patients with early cardiovascular-kidney-metabolic (CKM) and to evaluate their cumulative predictive value.

**Methods:**

Publicly available data from National Health and Nutrition Examination Survey (NHANES) 2007–2018 were used, including 10,201 participants aged 20 years or older who met the criteria for early-stage CKM. Multivariable logistic regression was employed to examine the independent associations of OBS and CDAI with frailty risk. RCS models were used to analyze non-linear relationships and threshold effects. Stepwise logistic regression, the DeLong test, and Net reclassification improvement/Integrated discrimination improvement (NRI/IDI) metrics were applied to compare the predictive performance and cumulative effects of the two indices.

**Results:**

Among the 10,201 early-stage CKM patients (mean age 46.30 [0.30] years; 51.40% female), multivariable logistic regression revealed that after adjusting for potential confounders, Quartile-based analyses indicated clear dose–response relationships: compared to the lowest quartile, the highest quartile of OBS was associated with a 54% lower risk of frailty (OR = 0.46, 95% CI: 0.36–0.60), and the highest quartile of CDAI was associated with a 39% lower risk (OR = 0.61, 95% CI: 0.50–0.74). Restricted cubic spline (RCS) analyses demonstrated significant non-linear associations for both OBS and CDAI with frailty risk (*P* for non-linearity = 0.002 and <0.001, respectively). An OBS threshold of approximately 9.05 was identified, beyond which frailty risk declined substantially. For CDAI, the threshold was about −2.39, below which frailty risk dropped markedly, while above this threshold, the risk plateaued with little further reduction. Cumulative effect analysis showed that the combined OBS and CDAI model (AUC = 0.577) did not offer a significant improvement over models including OBS alone (AUC = 0.577) or CDAI alone (AUC = 0.565), as indicated by DeLong test results (all *p* > 0.05). Additional analyses using continuous NRI and IDI metrics further confirmed the lack of significant additive effect when combining the two indices.

**Conclusion:**

This study found that both the OBS and the CDAI independently serve as protective factors against frailty in patients with early-stage CKM, each displaying a non-linear inverse association with clearly defined threshold effects.

## 1 Introduction

Cardiovascular-kidney-metabolic syndrome represents a multisystem disease spectrum encompassing the continuum of cardiovascular disease, chronic kidney disease, and metabolic disorders (1, 2). Early-stage CKM is defined as a state of mild organ dysfunction that does not yet meet the criteria for clinical diagnosis, and is characterized by subclinical manifestations such as mild endothelial dysfunction, microalbuminuria, and glucose metabolism abnormalities (3). Global epidemiological data indicate that with the increasing adoption of Western lifestyles and population aging, the prevalence of early-stage CKM is rising significantly, affecting 15–20% of individuals over 40 years old and up to 30–35% of the elderly. Prospective cohort research by Jankowski et al. (4) demonstrated that patients with early-stage CKM have a 2.8-fold higher risk (95% CI: 2.1–3.7, *p* < 0.001) of progressing to overt clinical disease within 5 years compared to controls.

Frailty is a distinct geriatric syndrome characterized by increased vulnerability to stressors and reduced physiological reserve, and has been closely linked to CKM (5). According to the phenotype defined by Fried et al. (6), frailty covers multiple domains including unintentional weight loss, fatigue, reduced physical activity, slower walking speed, and decreased grip strength. The prevalence of frailty is significantly higher among individuals with early-stage CKM than in age-matched healthy controls (18.7% vs. 8.3%, *p* < 0.001) (7). More concerningly, a meta-analysis by Walker et al. (7) found that frail individuals have a 2.54-fold increased risk of all-cause mortality (95% CI: 1.85–3.46) and a 1.82-fold higher hospitalization rate (95% CI: 1.56–2.15) over 5 years, along with significantly reduced quality of life (8). Therefore, identifying and intervening in risk factors for frailty among early CKM patients holds important clinical preventive value.

Oxidative stress is widely considered a key pathophysiological mechanism linking CKM with frailty (9, 10). At the molecular level, excessive production of reactive oxygen species and free radicals overwhelms endogenous antioxidant defenses, leading to lipid peroxidation, protein oxidative modifications, and DNA damage. These processes subsequently trigger inflammatory cascades and impair multiorgan function (11). Studies by Wang et al. (12) using oxidative markers such as advanced oxidation protein products and malondialdehyde revealed a significant positive correlation between oxidative stress levels and the frailty index (*r* = 0.37, *p* < 0.001). This association remained significant after adjusting for age, sex, and comorbidities (OR = 1.42, 95% CI: 1.18–1.71) (12). Notably, in early-stage CKM, elevations in oxidative stress markers precede clinical symptoms, suggesting their potential role as early indicators of disease progression and functional decline (13).

The Oxidative Balance Score (OBS) is a composite index that evaluates an individual’s overall oxidative-antioxidative status by integrating pro-oxidant factors (such as smoking, saturated fat intake, and excessive iron) and antioxidant factors (such as fruit and vegetable intake, vitamin E/C supplementation, and physical activity) (14). Multiple studies have demonstrated that OBS shows a significant inverse association with various chronic disease risks. For example, Ilori et al. (15), in a long-term study of 10,881 middle-aged and elderly participants, found that those in the highest OBS quartile had a 27% lower risk of cardiovascular events and a 25% lower risk of end-stage renal disease compared to those in the lowest quartile. Goodman et al. (16) observed that every 5-point increase in OBS corresponded to a 13% reduction in type 2 diabetes risk. These findings underscore the central role of oxidative balance in the CKM disease spectrum.

The Composite Dietary Antioxidant Index (CDAI), on the other hand, focuses on evaluating the cumulative effect of dietary antioxidants, including micronutrients such as vitamins C and E, carotenoids, flavonoids, selenium, and zinc (17). Unlike traditional assessments focusing on single nutrients, CDAI accounts for the synergistic effects among multiple dietary antioxidants within food matrices (18). Although both OBS and CDAI have been broadly studied in relation to chronic disease risk, their potential additive effect on frailty risk among patients with early-stage CKM remains a crucial knowledge gap (19). Theoretically, OBS and CDAI may modulate oxidative balance through different yet complementary mechanisms: OBS incorporates a broader range of lifestyle and non-dietary factors, while CDAI provides a detailed assessment of dietary antioxidant capacity (20). However, it remains unclear whether synergistic, antagonistic, or non-linear dose–response relationships exist between these two indices (21). Studies by Lakkur et al. (22) found that OBS was inversely related to systemic inflammatory markers such as CRP and IL-6, while Brighenti et al. (23) confirmed an association between CDAI and the DNA oxidative damage marker 8-OHdG, suggesting that OBS and CDAI may influence frailty risk through distinct intermediary pathways.

Importantly, interventional research targeting the early stages of CKM—a period recognized as the ‘golden window’ during which pathological changes remain reversible and preventive measures are most effective—remains scarce. Traditional research has primarily focused on individuals already diagnosed with chronic diseases, neglecting intervention opportunities at the preclinical stage. Therefore, clarifying the additive effect of OBS and CDAI on frailty risk among early-stage CKM patients will not only enhance our understanding of oxidative stress’s role in frailty development but also identify new targets for clinical prevention. This study aims to explore the potential additive effects of OBS and CDAI on frailty risk in early-stage CKM patients, analyze their dose–response and threshold effects, and assess heterogeneity across subgroups. The findings are expected to provide scientific evidence for formulating precise nutritional and lifestyle interventions, with the ultimate goal of improving long-term outcomes and reducing disease progression and functional decline in early CKM patients.

## 2 Methods

### 2.1 Data sources and study design

This study utilized publicly available data from the NHANES conducted between 2007 and 2018. NHANES is a nationwide, cross-sectional survey carried out by the National Center for Health Statistics of the U. S. Centers for Disease Control and Prevention. It employs a multistage, stratified probability sampling design to assess the health and nutritional status of the non-institutionalized U. S. population (24). For this study, we utilized six continuous cycles of NHANES from 2007–2008 to 2017–2018. This period was selected to ensure consistency in dietary data collection methodology, as all cycles used the interviewer-administered AMPM 24-h recall. Later cycles (2019–2020) were excluded due to the implementation of a different dietary data collection platform and the severe disruption of data collection caused by the COVID-19 pandemic, which resulted in a non-representative sample and potential confounding from pandemic-related lifestyle shifts. All NHANES participants provided written informed consent, and the study protocol was approved by the CDC Institutional Review Board.

### 2.2 Study subjects

The staging of CKM in this study was defined according to the latest consensus, incorporating data on medical history, laboratory results, and physical examination indicators collected by NHANES (25, 26). CKM Stage 0: Healthy individuals without underlying diseases or risk factors. CKM Stages 1–3: Encompasses individuals with metabolic syndrome, early kidney damage, and/or early cardiovascular dysfunction. Specific indicators include hypertension, diabetes, obesity, eGFR, and urinary albumin levels. CKM Stage 4: Clinical cardiovascular disease, including coronary heart disease, heart failure, stroke, peripheral arterial disease, and atrial fibrillation occurring on a CKM background. Early-stage CKM includes all patients in stages 0–3 (27). In accordance with the American Heart Association’s conceptual framework for CKM syndrome, Stage 0 was included to establish a reference group of individuals with no risk factors, representing the ideal state of cardiovascular-kidney-metabolic health. This allows for the assessment of risk gradients across the full clinical spectrum and aligns with the model’s focus on primordial prevention. Detailed criteria are provided in Supplementary Table S1.

**Figure 1:**
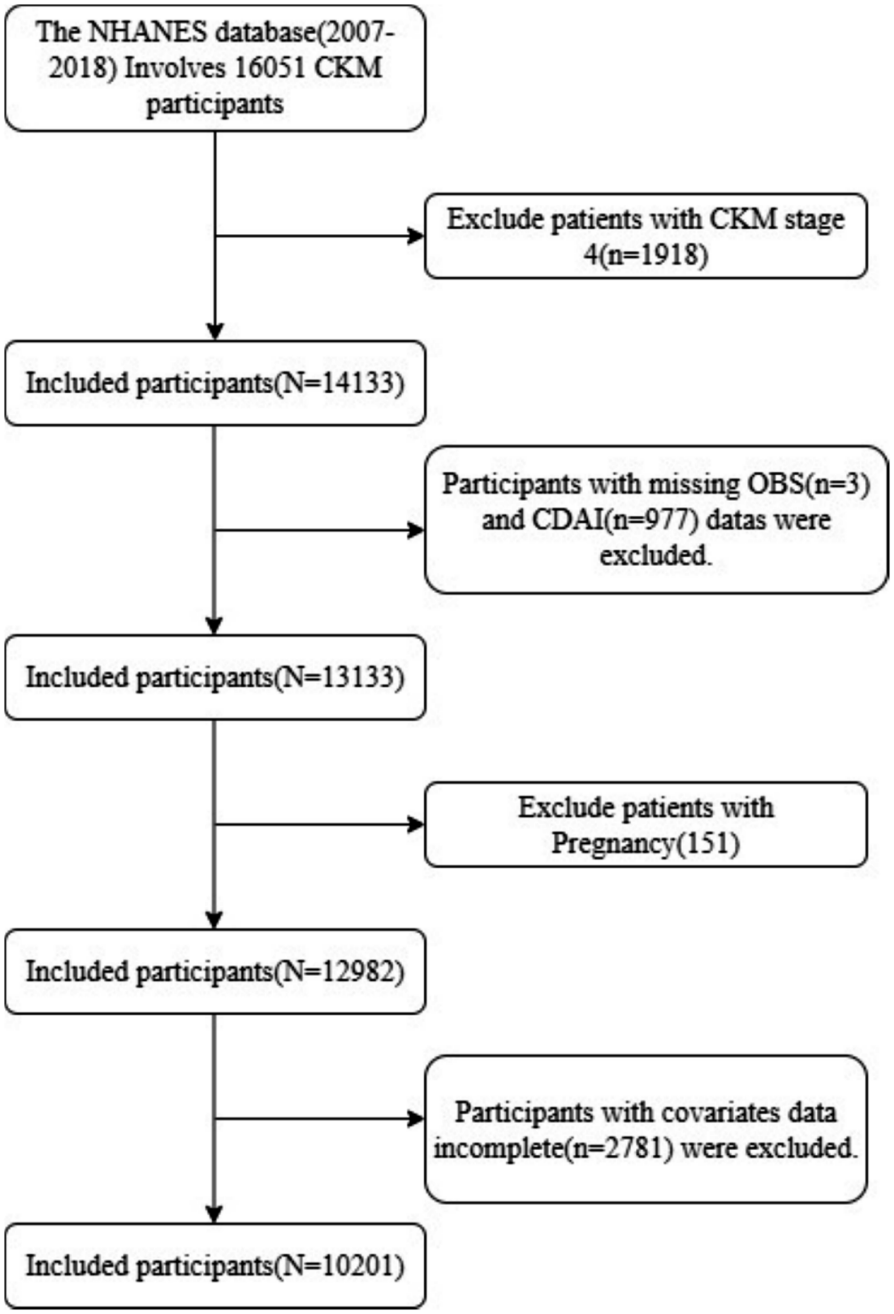
Study flow chart.

This study included adult participants aged 20 years and older from the NHANES 2007–2018 dataset. The exclusion criteria were as follows: (1) individuals in CKM Stage 4 (*n* = 1918); (2) pregnant women (*n* = 151); (3) participants lacking OBS (*n* = 3) and CDAI data (*n* = 977); (4) those missing essential data for frailty assessment (*n* = 0); and (5) those with missing data for other covariates (*n* = 2,781). Ultimately, a total of 10,201 eligible participants were included in the analysis (Figure 1).

### 2.3 Assessment of oxidative balance score

The Oxidative Balance Score (OBS) was constructed based on established methodologies from prior epidemiological studies (16, 28), integrating 20 components that represent both antioxidant and pro-oxidant exposures. These comprised 16 dietary factors assessed via the first 24-h dietary recall and 4 lifestyle factors. The 15 antioxidant components included: dietary fiber, carotenoids (as retinol equivalents), riboflavin, niacin, vitamin B6, total folate, vitamin B12, vitamin C, vitamin E (as *α*-tocopherol equivalents), calcium, magnesium, zinc, copper, selenium, and physical activity level. The 5 pro-oxidant components included: total fat intake, iron intake, body mass index (BMI), alcohol consumption, and smoking exposure (quantified by serum cotinine levels).

The scoring logic for each component was assigned based on sex-specific quartiles (Q1–Q4) of their distribution within the NHANES population: For each antioxidant component, participants received a score of 0, 1, or 2, corresponding to the first (lowest), second/third, or fourth (highest) quartile of intake/level, respectively. For each pro-oxidant component, the scoring was reversed. Participants received a score of 2, 1, or 0, corresponding to the first (lowest), second/third, or fourth (highest) quartile of intake/level, respectively. This reversal reflects the hypothesized harmful (pro-oxidative) effect of higher levels of these exposures. The individual scores for all 20 components were summed to create a total OBS for each participant. A higher total OBS indicates a greater overall predominance of antioxidant over pro-oxidant exposures.

### 2.4 Assessment of comprehensive dietary antioxidant index

In the NHANES study, trained interviewers collected information on participants’ intake of dietary antioxidants and other food components using two 24-h dietary recall interviews. The first interview was typically conducted at a mobile examination center, while the second recall was completed by telephone 3–10 days later. Participants were required to recall and report in detail all foods and beverages consumed in the previous 24 h, as well as any dietary supplements used, including specific dosages, frequency, and duration of use (29). The CDAI focuses on six food-derived antioxidants: vitamins A, C, and E, zinc, selenium, and carotenoids, and does not take into account antioxidant contributions from supplements, medications, or drinking water. CDAI is calculated by subtracting the overall mean intake from an individual’s actual intake for each antioxidant, then dividing by the standard deviation to obtain a standardized value.


$$\text{CDAI}=\sum_{i=1}^{6}\frac{\text{Individual Intake}-\text{Mean}}{\mathrm{SD}}.$$

### 2.5 Assessment of frailty

Frailty was defined in this study using a frailty index with a cutoff value of ≥0.21 (30). The index, originally developed by Sabbah (31) based on the approach described by Searle et al. (32), includes 49 criteria spanning multiple domains related to frailty, such as cognitive function, mood (depression), activities of daily living, physical performance, chronic illness, self-rated health, healthcare utilization, and laboratory parameters. Supplementary Table S2 provides the detailed criteria. Each item is scored from 0 to 1 according to its severity, and the individual’s frailty index is calculated by dividing the total score by the number of items assessed. Frailty was assessed using a 49-item cumulative deficit Frailty Index (FI), constructed in accordance with the validated methodology for the NHANES database (33). Consistent with this protocol, participants were required to have non-missing data for at least 80% (≥39 out of 49) of the deficit items to be included in the analysis. This ensures the reliability of the index for each individual assessed. For these included participants, the FI was calculated as the proportion of deficits present out of the total number of non-missing items. No data imputation was performed for the remaining missing items; this approach is a standard practice in FI construction to avoid introducing bias and is validated against clinical outcomes (32–34). Constructed in accordance with the validated methodology specific to the NHANES database (33). Participants with an FI ≥ 0.21 were classified as frail. This threshold has been demonstrated to optimally identify individuals at the highest risk for mortality and adverse health outcomes within the NHANES population (33).

### 2.6 Covariates

To control for confounding effects, a variety of demographic and clinical characteristics were included in the analysis. Demographic characteristics: age, sex, race/ethnicity, education level, income (poverty income ratio, PIR), and marital status. Lifestyle factors: smoking status (never, former, current), alcohol consumption (never, former, mild drinking, moderate drinking, and heavy drinking). Anthropometric indicator: BMI. Medical history: diabetes and hypertension. Medication use: antihypertensive agents, antidiabetic agents, and lipid-lowering agents. Renal function indicators: serum creatinine, UA, BUN, and eGFR. Lipid profile: TC, HDL, LDL, and TG. Glucose metabolism: FPG and HbA1c.

### 2.7 Statistical analysis

Participant characteristics were described according to frailty status (frail vs. non-frail groups). Continuous variables were presented as means (standard error), while categorical variables were expressed as percentages (standard error). Group differences were compared using *t*-tests, Mann–Whitney *U* tests, chi-square tests, or Fisher’s exact tests, as appropriate. All analyses considered the NHANES complex sampling design and weighted using MEC sample weights (WTMEC2YR/6). Multivariable logistic regression models were used to assess the independent associations of OBS and CDAI with frailty risk. Three hierarchical models were constructed: Model 1: unadjusted; Model 2: adjusted for age, sex, and race/ethnicity; Model 3: further adjusted for hypertension, diabetes, use of antidiabetic, antihypertensive, and antihyperlipidemic medications, BMI, eGFR, HbA1c, TG, and HDL. Results were reported as odds ratios and 95% confidence intervals. Non-linear relationships were explored using RCS with four knots placed at the 5th, 35th, 65th, and 95th percentiles of the CDAI and OBS distributions, as per standard methodological recommendations. The significance of any non-linear relationship, as well as possible threshold effects, was evaluated using the likelihood ratio test. Multicollinearity among the covariates in the fully-adjusted model (Model 3) was assessed using the variance inflation factor (VIF). All individual VIF values were below 5, indicating that multicollinearity was not a substantive concern in our analysis (Supplementary Table S3).

To assess the additive predictive value of combining OBS and CDAI for frailty risk, the following analytic steps were taken: ① Model construction: single-marker models for OBS and CDAI, and a combined OBS + CDAI model; ② Evaluation of predictive performance: AUC was calculated for each model, differences in AUCs were compared using the DeLong test, and the NRI and IDI were computed to quantify improvements in prediction gained by adding OBS and CDAI. Potential interactions of OBS and CDAI with age, sex, hypertension, and diabetes on outcomes were examined by including interaction terms in the model and conducting stratified subgroup analyses as appropriate. All statistical analyses were performed using R software (version 4.3.2), and a two-sided *p*-value < 0.05 was considered statistically significant.

## 3 Results

### 3.1 Basic characteristics of study subjects

The final analysis included 10,201 participants who met the criteria for early-stage CKM, with a mean age of 46.30 (0.30) years, and 51.40% of whom were female. A total of 1,643 participants (16.11%) were classified as frail. Table 1 presents the baseline characteristics of participants stratified by frailty status. Compared to the non-frail group, participants in the frail group were older (53.98 [0.53] vs. 45.10 [0.32] years, *p* < 0.001) and had a higher proportion of females (66.98% vs. 48.98%, *p* < 0.001). They also had lower levels of education and income (*p* < 0.001). The frail group exhibited a higher body mass index (31.4[0.72] vs. 29.3[0.61]kg/m^2^, *p* < 0.001) and were more likely to have hypertension (66.53% vs. 30.16%, *p* < 0.001) and diabetes (34.92% vs. 10.67%, *p* < 0.001). Additionally, both the OBS and CDAI were significantly lower in the frail group compared to the non-frail group (OBS: 17.57 [0.30] vs. 19.89 [0.19], *p* < 0.001; CDAI: 0.12 [0.13] vs. 0.92 [0.07], *p* < 0.001).

**Table 1:** Frailty patients versus non-frailty patients general clinical characteristics.

Variables	Total (*n* = 10,201)	Non-frailty (*n* = 8,558)	Frailty (*n* = 1,643)	*p*-value
Age, mean (SE)	46.30(0.30)	45.10(0.32)	53.98(0.53)	<0.001
Creatinine, mean (SE)	76.47(0.37)	75.72(0.28)	81.29(2.22)	0.020
UA, mean (SE)	324.27(1.24)	323.16(1.23)	331.37(3.37)	0.020
BUN, mean (SE)	4.77(0.03)	4.70(0.03)	5.21(0.08)	<0.001
TG, mean (SE)	1.29(0.01)	1.26(0.01)	1.52(0.04)	<0.01
HDL, mean (SE)	1.41(0.01)	1.42(0.01)	1.36(0.02)	0.003
BMI, mean (SE)	28.93(0.11)	28.45(0.11)	32.03(0.28)	<0.001
TC, mean (SE)	4.99(0.02)	4.98(0.02)	5.03(0.04)	0.340
LDL, mean (SE)	2.98(0.01)	2.99(0.01)	2.97(0.03)	0.520
eGFR, mean (SE)	96.36(0.40)	97.47(0.43)	89.29(0.81)	<0.001
FPG, mean (SE)	5.84(0.02)	5.74(0.02)	6.51(0.06)	<0.001
HbA1c, mean (SE)	5.58(0.01)	5.51(0.01)	6.05(0.04)	<0.001
OBS, mean (SE)	19.58(0.18)	19.89(0.19)	17.57(0.30)	<0.001
CDAI, mean (SE)	0.81(0.07)	0.92(0.07)	0.12(0.13)	<0.001
Sex, % (SE)				<0.001
Female	51.40(0.02)	48.98(0.61)	66.98(1.59)	
Male	48.60(0.01)	51.02(0.61)	33.02(1.59)	
Race, % (SE)				<0.001
Mexican American	8.36(0.01)	8.64(0.75)	6.59(0.78)	
Non-Hispanic Black	9.72(0.01)	9.09(0.67)	13.74(1.26)	
Non-Hispanic White	69.28(0.03)	69.51(1.42)	67.78(1.89)	
Other	12.65(0.01)	12.76(0.74)	11.89(1.04)	
Marital, % (SE)				<0.001
Divorced	10.64(0.01)	9.81(0.49)	16.03(1.19)	
Married	55.39(0.02)	56.54(1.07)	48.07(1.91)	
Never married	18.89(0.01)	19.72(0.79)	13.53(1.02)	
Other	15.07(0.01)	13.94(0.57)	22.37(1.28)	
Education, % (SE)				<0.001
High school or equivalent	22.56(0.01)	21.80(0.84)	27.39(1.58)	
Less than high school	14.10(0.01)	12.78(0.68)	22.59(1.26)	
Some college or above	63.35(0.02)	65.42(1.20)	50.02(1.99)	
Smoke, % (SE)				<0.001
Former	24.66(0.01)	24.22(0.77)	27.50(1.60)	
Never	56.71(0.02)	58.78(0.85)	43.44(1.73)	
Now	18.63(0.01)	17.00(0.63)	29.06(1.82)	
Alcohol, % (SE)				<0.001
Former	13.61(0.01)	11.95(0.52)	24.30(1.37)	
Heavy	21.35(0.01)	21.70(0.67)	19.04(1.30)	
Mild	37.10(0.01)	38.34(0.95)	29.13(1.92)	
Moderate	18.09(0.01)	18.50(0.58)	15.49(1.25)	
Never	9.85(0.01)	9.51(0.59)	12.04(1.00)	
Hypertension, % (SE)				<0.001
No	64.94(0.02)	69.84(0.80)	33.47(1.37)	
Yes	35.06(0.01)	30.16(0.80)	66.53(1.37)	
Diabetes, % (SE)				<0.001
Borderline	17.25(0.01)	16.81(0.60)	20.07(1.28)	
No	68.82(0.02)	72.53(0.76)	45.01(1.61)	
Yes	13.94(0.01)	10.67(0.47)	34.92(1.31)	
Antidiabetic, % (SE)				<0.001
No	92.36(0.03)	94.86(0.34)	76.34(1.29)	
Yes	7.64(0.00)	5.14(0.34)	23.66(1.29)	
Antihypertension, % (SE)				<0.001
No	75.51(0.02)	80.35(0.71)	44.44(1.60)	
Yes	24.49(0.01)	19.65(0.71)	55.56(1.60)	
Antihyperlipidemic, % (SE)				<0.001
No	84.55(0.02)	87.25(0.56)	67.23(1.56)	
Yes	15.45(0.01)	12.75(0.56)	32.77(1.56)	
PIR, % (SE)				<0.001
<1.3	20.68(0.01)	18.67(0.79)	33.54(1.70)	
>3.5	43.95(0.02)	46.29(1.21)	28.95(1.67)	
1.3–3.5	35.38(0.01)	35.04(0.91)	37.51(1.62)	
OBS. Q, % (SE)				<0.001
Q1	22.05(0.01)	20.81(0.78)	30.01(1.80)	
Q2	26.93(0.01)	26.49(0.73)	29.70(1.67)	
Q3	26.03(0.01)	26.35(0.70)	23.99(1.69)	
Q4	25.00(0.01)	26.35(0.79)	16.30(1.45)	
CDAI. Q, % (SE)				<0.001
Q1	22.08(0.01)	20.94(0.66)	29.36(1.46)	
Q2	24.42(0.01)	24.30(0.53)	25.15(1.54)	
Q3	26.49(0.01)	27.01(0.60)	23.15(1.59)	
Q4	27.02(0.01)	27.74(0.65)	22.34(1.50)	

Date are presented as mean (SE) or *n* (%); PIR, poverty income ratio; BMI, body mass index; FPG, fasting plasma glucose; HbA1c, glycosylated hemoglobin; UA, uric acid; BUN, blood urea nitrogen; TG, triglyceride; TC, total cholesterol; HDL, high density lipoprotein; LDL, low density lipoprotein; eGFR, estimated glomerular filtration rate; OBS, oxidative balance score; CDAI, composite dietary antioxidant index.

### 3.2 Analysis of association between OBS, CDAI and risk of frailty

Table 2 presents the results of multivariable logistic regression analyses evaluating the associations between OBS, CDAI, and frailty risk. In the unadjusted model, each 1-unit increase in OBS was associated with a 3% reduction in frailty risk (OR = 0.97, 95% CI: 0.96–0.98, *p* < 0.001), while each 1-unit increase in CDAI corresponded to a 6% reduction (OR = 0.94, 95% CI: 0.92–0.97, *p* < 0.001). After full adjustment (Model 3), each 1-unit increase in OBS remained significantly associated with a 3% decrease in frailty risk (OR = 0.97, 95% CI: 0.96–0.98, *p* < 0.001), and each 1-unit increase in CDAI with a 4% decrease (OR = 0.96, 95% CI: 0.94–0.98, *p* < 0.001). These results indicate that both OBS and CDAI are independent protective factors against frailty in patients with early-stage CKM syndrome. Further analyses using quartile groupings (Q1–Q4) revealed a clear dose–response relationship. Compared with participants in the lowest quartile (Q1) of OBS, those in the highest quartile (Q4) had a 54% lower frailty risk (OR = 0.46, 95% CI: 0.36–0.60, *p* < 0.001; *P* for trend < 0.001). Similarly, participants in the highest quartile of CDAI exhibited a 39% lower risk compared to those in the lowest quartile (OR = 0.61, 95% CI: 0.50–0.74, *p* < 0.001; *P* for trend < 0.001). A sensitivity analysis excluding individuals in CKM Stage 0 was performed to assess the robustness of the observed associations (Supplementary Table S4).

**Table 2:** Association of OBS, CDAI and risk of frailty in early CKM patients.

Variables	Model 1		Model 2		Model 3	
	OR (95%CI)	*P*	OR (95%CI)	*P*	OR (95%CI)	*P*
OBS	0.97(0.96,0.98)	<0.001	0.96(0.96,0.97)	<0.001	0.97(0.96,0.98)	<0.001
OBSQ						
Q1	Ref	Ref	Ref	Ref	Ref	Ref
Q2	0.78(0.64,0.95)	0.010	0.68(0.55,0.85)	<0.001	0.66(0.52,0.84)	0.001
Q3	0.63(0.50,0.80)	<0.001	0.56(0.43,0.71)	<0.001	0.57(0.44,0.74)	<0.001
Q4	0.43(0.34,0.54)	<0.001	0.39(0.30,0.50)	<0.001	0.46(0.36,0.60)	<0.001
*P* for trend	<0.001		<0.001		<0.001	
CDAI	0.94(0.92,0.97)	<0.001	0.95(0.93,0.97)	<0.001	0.96(0.94,0.98)	<0.001
CDAIQ						
Q1	Ref	Ref	Ref	Ref	Ref	Ref
Q2	0.74(0.61,0.89)	0.002	0.74(0.61,0.90)	0.003	0.72(0.58,0.90)	0.004
Q3	0.61(0.50,0.75)	<0.001	0.64(0.52,0.79)	<0.001	0.63(0.51,0.78)	<0.001
Q4	0.57(0.48,0.69)	<0.001	0.62(0.51,0.75)	<0.001	0.61(0.50,0.74)	<0.001
*P* for trend	<0.001		<0.001		<0.001	

OR, odds ratio; CI, confidence interval; Ref, reference.

Model 1: No adjustments made.

Model 2: Adjusted for age, sex, race.

Model 3: Adjusted for age, sex, race, hypertension, diabetes, antidiabetic, antihypertension, antihyperlipidemic, BMI, eGFR, HbA1c, TG, HDL.

### 3.3 Non-linear association of OBS, CDAI and risk of frailty and threshold effect

**Figure 2:**
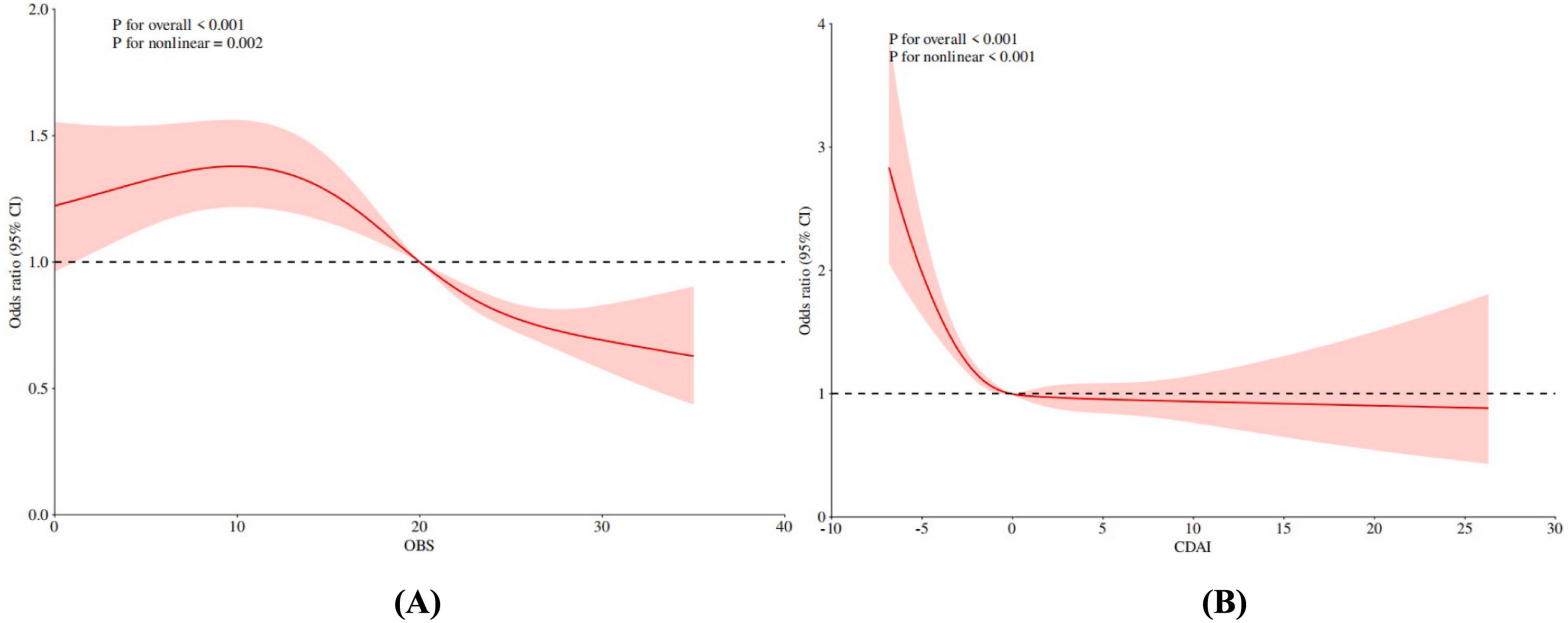
Non-linear association RCS analysis of OBS, CDAI and risk of frailty in early CKM patients. **(A)** OBS, **(B)** CDAI. OBS, oxidative balance score; CDAI, composite dietary antioxidant index.

Figure 2 presents the dose–response relationships between OBS, CDAI, and frailty risk, as evaluated using restricted cubic spline (RCS) analysis. A significant non-linear association was observed for OBS (*P* for non-linearity = 0.002), with an identified threshold value of 9.05. Frailty risk initially increased with rising OBS values but declined steadily beyond this inflection point (Table 3). Similarly, CDAI exhibited a significant non-linear relationship with frailty risk (*p* < 0.001). Frailty risk decreased rapidly with increasing CDAI at lower values, after which the rate of decline attenuated, indicating a plateau effect. The threshold for CDAI was approximately −2.39; beyond this value, further increases in CDAI were associated with a markedly diminished reduction in frailty risk, suggesting a protective plateau (Table 3). These findings indicate a non-linear threshold effect in the associations of both OBS and CDAI with frailty risk. Specifically, improvements in oxidative balance or dietary antioxidant capacity below their respective thresholds may lead to substantial reductions in frailty risk, whereas the marginal benefit of additional increases beyond these values appears to diminish.

**Table 3:** Threshold effect analysis of OBS, CDAI and risk of frailty in early CKM patients.

Outcome	Effect	*P*
OBS		
Model 1 Fitting model by standard linear regression	0.97 (0.97–0.98)	<0.001
Model 2 Fitting model by two-piecewise linear regression		
Inflection point	9.05	
<9.05	1.04 (0.99–1.09)	0.168
≥9.05	0.96 (0.95–0.97)	<0.001
*P* for likelihood test		<0.001
CDAI		
Model 1 Fitting model by standard linear regression	0.96 (0.94–0.97)	<0.001
Model 2 Fitting model by two-piecewise linear regression		
Inflection point	−2.39	
<−2.39	0.79 (0.71–0.88)	<0.001
≥ − 2.39	0.98 (0.96–1.00)	0.096
*P* for likelihood test		<0.001

OBS, oxidative balance score; CDAI, composite dietary antioxidant index.

### 3.4 Additive effect analysis of OBS and CDAI on frailty risk prediction

As detailed in Table 4, the cumulative predictive effects of OBS and CDAI—both individually and in combination—on frailty risk were evaluated among all participants. Patients with high OBS (≥9.05) and high CDAI (≥−2.39) exhibited a significantly reduced risk of frailty (OR = 0.49, 95% CI: 0.39–0.63, *p* < 0.001). This association remained statistically significant in subsequent models adjusted for covariates, with only marginal changes in effect size: Model 1 (OR = 0.48, 95% CI: 0.39–0.61, *p* < 0.001) and Model 2 (OR = 0.47, 95% CI: 0.37–0.60, *p* < 0.001).

**Table 4:** Association of OBS + CDAI combination with mortality in the overall population and various subgroups.

Variables	Low OBS and low CDAI (OBS < 9.05 and CDAI < −2.39)	Low OBS and high CDAI (OBS < 9.05 and CDAI ≥ −2.39)	High OBS and low CDAI (OBS ≥ 9.05 and CDAI < −2.39)	High OBS and high CDAI (OBS ≥ 9.05 and CDAI ≥ −2.39)	*P*-value
Overall					
Model 1	Ref	0.51(0.39,0.67)	0.69(0.52,0.92)	0.48(0.39,0.61)	<0.001
Model 2	Ref	0.63(0.46,0.87)	0.65(0.48,0.88)	0.47(0.37,0.60)	<0.001
Model 3	Ref	0.66(0.46,0.93)	0.71(0.52,0.96)	0.49(0.39,0.63)	<0.001
Male					
Model 1	Ref	0.50(0.32,0.78)	0.54(0.32,0.93)	0.51(0.35,0.75)	0.020
Model 2	Ref	0.60(0.37,0.98)	0.55(0.31,0.97)	0.53(0.35,0.80)	0.010
Model 3	Ref	0.60(0.36,1.00)	0.55(0.30,1.00)	0.54(0.36,0.81)	0.020
Female					
Model 1	Ref	0.62(0.40,0.95)	0.73(0.49,1.09)	0.47(0.33,0.67)	<0.001
Model 2	Ref	0.68(0.44,1.05)	0.70(0.46,1.07)	0.45(0.31,0.65)	<0.001
Model 3	Ref	0.69(0.42,1.14)	0.78(0.51,1.19)	0.46(0.31,0.69)	<0.001
Age < 65					
Model 1	Ref	0.47(0.33,0.66)	0.72(0.50,1.03)	0.48(0.36,0.64)	<0.001
Model 2	Ref	0.54(0.37,0.78)	0.72(0.50,1.03)	0.51(0.38,0.68)	<0.001
Model 3	Ref	0.53(0.34,0.84)	0.76(0.53,1.11)	0.47(0.35,0.64)	<0.001
Age ≥ 65					
Model 1	Ref	1.07(0.53,2.16)	0.60(0.35,1.01)	0.50(0.33,0.76)	<0.001
Model 2	Ref	1.10(0.54,2.24)	0.59(0.34,1.00)	0.52(0.33,0.80)	<0.001
Model 3	Ref	1.50(0.73,3.07)	0.64(0.36,1.13)	0.63(0.42,0.93)	0.002
Hypertension					
Model 1	Ref	0.71(0.46,1.10)	0.88(0.59,1.31)	0.61(0.44,0.84)	0.003
Model 2	Ref	0.80(0.51,1.27)	0.82(0.54,1.25)	0.62(0.44,0.87)	0.003
Model 3	Ref	0.80(0.50,1.30)	0.81(0.53,1.25)	0.58(0.42,0.80)	<0.001
Non-hypertension					
Model 1	Ref	0.37(0.23,0.60)	0.54(0.36,0.81)	0.37(0.27,0.51)	<0.001
Model 2	Ref	0.44(0.27,0.73)	0.51(0.34,0.76)	0.36(0.25,0.50)	<0.001
Model 3	Ref	0.47(0.28,0.80)	0.57(0.37,0.88)	0.40(0.27,0.58)	<0.001
Diabetes					
Model 1	Ref	0.59(0.34,1.03)	0.62(0.40,0.95)	0.62(0.41,0.94)	0.130
Model 2	Ref	0.58(0.33,1.03)	0.53(0.36,0.79)	0.58(0.40,0.86)	0.090
Model 3	Ref	0.57(0.32,1.03)	0.53(0.34,0.84)	0.56(0.37,0.85)	0.070
Non-diabetes					
Model 1	Ref	0.54(0.34,0.85)	0.70(0.44,1.10)	0.40(0.28,0.56)	<0.001
Model 2	Ref	0.67(0.40,1.12)	0.68(0.42,1.10)	0.38(0.26,0.56)	<0.001
Model 3	Ref	0.75(0.43,1.28)	0.79(0.49,1.28)	0.42(0.29,0.62)	<0.001
Borderline-diabetes					
Model 1	Ref	0.48(0.26,0.88)	0.88(0.50,1.53)	0.72(0.42,1.23)	0.730
Model 2	Ref	0.59(0.31,1.11)	0.83(0.46,1.50)	0.70(0.40,1.22)	0.420
Model 3	Ref	0.48(0.26,0.91)	0.78(0.41,1.47)	0.61(0.34,1.09)	0.290

Model 1: No adjustments made.

Model 2: Adjusted for age, sex, race.

Model 3: Adjusted for age, sex, race, hypertension, diabetes, antidiabetic, antihypertension, antihyperlipidemic, BMI, eGFR, HbA1c, TG, HDL.

OBS, oxidative balance score; CDAI, composite dietary antioxidant index.

**Figure 3:**
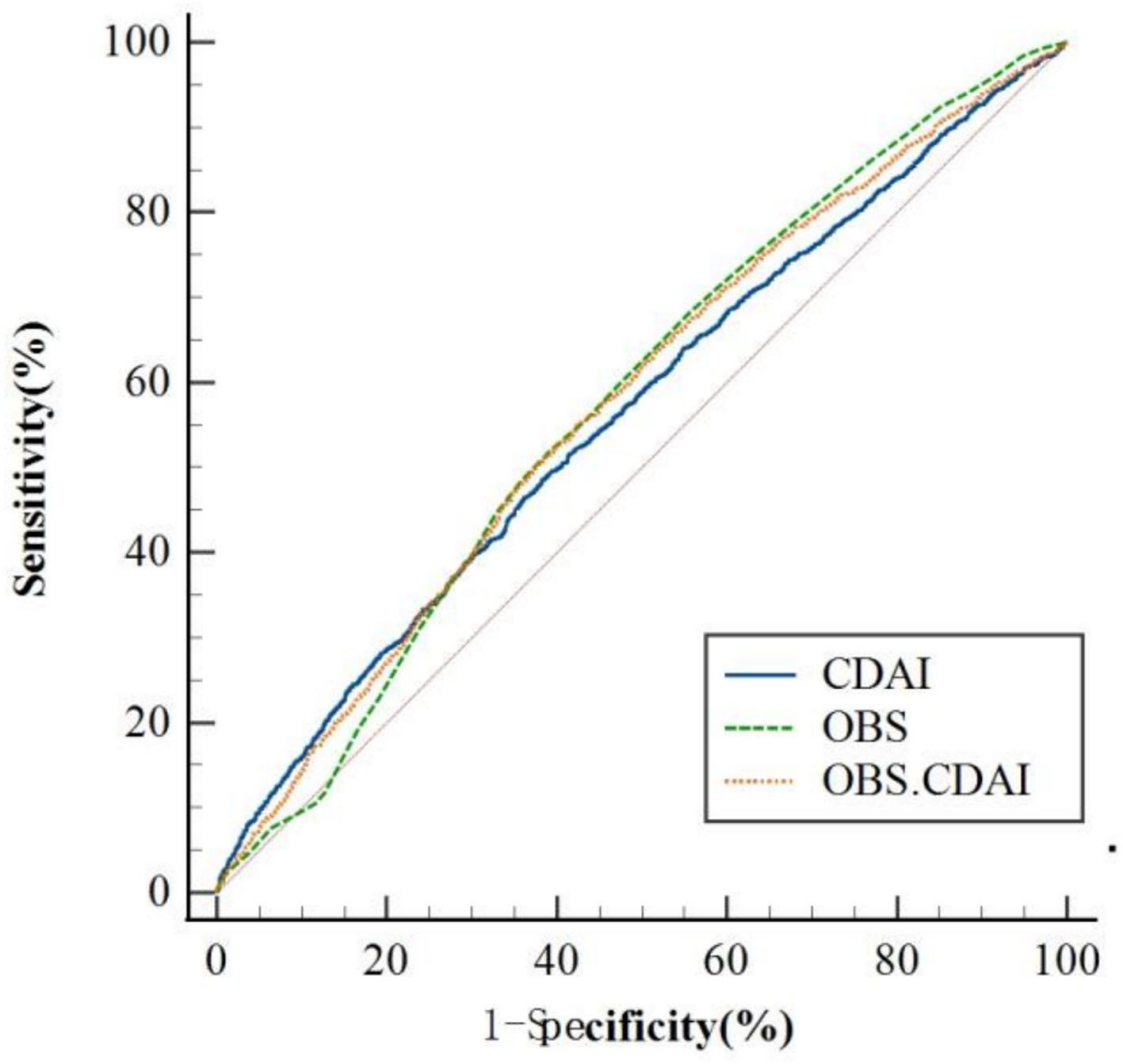
ROC analysis of OBS, CDAI, and their combination for risk prediction of frailty in early CKM patients; OBS, oxidative balance score; CDAI, composite dietary antioxidant index.

As presented in Table 5 and Figure 3, the area under the receiver operating characteristic curve (AUC) was compared across models: OBS alone (AUC = 0.577, 95% CI: 0.568–0.587), CDAI alone (AUC = 0.565, 95% CI: 0.555–0.575), and the combined OBS + CDAI model (AUC = 0.577, 95% CI: 0.568–0.587). The DeLong test revealed that the combined model did not yield a significant improvement in predictive performance compared to the OBS-alone model (AUC difference = 0.000, *p* > 0.05). However, a small but statistically significant increase in AUC was observed for the combined model relative to the CDAI-alone model (ΔAUC = 0.012, *p* = 0.011).

**Table 5:** Predictive value of OBS, CDAI and their combination for risk of frailty in patients with early CKM.

Models	Harrell’s C-index	*P*-value	Adjusted Harrell’s C-index	*P*-value
OBS + CDAI vs. OBS	0.577(0.568–0.587) vs.0.577(0.568–0.587)	0.939	0.773(0.764–0.781) vs.0.773(0.764–0.781)	0.880
OBS + CDAI vs. CDAI	0.577(0.568–0.587) vs.0.565(0.555,0.575)	0.011	0.773(0.764–0.781) vs.0.770(0.762–0.778)	0.148

OBS, oxidative balance score; CDAI, composite dietary antioxidant index.

We further assessed incremental predictive value using net reclassification improvement (NRI) and integrated discrimination improvement (IDI). Compared with the OBS-only model, the combined OBS + CDAI model showed a continuous NRI of 0.0067 (95% CI: −0.0012 to 0.0131, *p* = 0.116) and an IDI of 0.0032 (95% CI: −0.0054 to 0.0118, *p* = 0.466). Similarly, when compared to the CDAI-only model, the continuous NRI was 0.0055 (95% CI: −0.0021 to 0.0103, *p* = 0.243) and the IDI was 0.0018 (95% CI: −0.0089 to 0.0125, *p* = 0.745). None of these improvements reached statistical significance (Table 6).

**Table 6:** Comparison of NRI and IDI for predictive value of OBS, CDAI and their combination for risk of frailty in early CKM patients.

Models	AUC (95%CI)	NRI (categorical)	*P*-value	IDI	*P*-value
OBS	0.577(0.568–0.587)	–	–	–	–
CDAI	0.565(0.555,0.575)	–	–	–	–
OBS + CDAI^#^	0.577(0.568-0.587)	0.4167(−0.1025–0.9359)	0.116	0.0032(−0.0054–0.0118)	0.466
OBS + CDAI^*^	0.577(0.568–0.587)	0.3155(−0.2141–0.845)	0.243	0.0018(−0.0089–0.0125)	0.745

^#^OBS + CDAI vs. OBS; ^*^OBS + CDAI vs. CDAI; OBS, oxidative balance score; CDAI, composite dietary antioxidant index.

### 3.5 Subgroup analysis

Stratified analyses demonstrated that the protective associations observed in the overall population remained consistent and statistically significant (*p* < 0.05) across all subgroups. As presented in Table 4, participants with both high OBS and high CDAI consistently exhibited the lowest risk of frailty within each subgroup including males and females, those younger than 65 and those 65 or older, as well as individuals with or without hypertension or diabetes. Furthermore, these subgroup analyses confirmed that no significant additive effect existed between OBS and CDAI in relation to frailty risk.

## 4 Discussion

This study systematically evaluated the associations and potential cumulative effects of the Oxidative Balance Score (OBS) and the Composite Dietary Antioxidant Index (CDAI) on frailty risk among patients with early-stage cardiovascular-kidney-metabolic (CKM) syndrome. The results demonstrated that both higher OBS and CDAI were independent protective factors against frailty and exhibited significant dose–response relationships. Their associations with frailty risk were non-linear, with clearly identifiable threshold effects. Although the combination of high OBS and high CDAI was significantly associated with reduced frailty risk, predictive model analyses did not indicate a significant cumulative effect. These findings provide new insights into the complex relationship between oxidative balance and frailty risk in early CKM patients.

We found that each 1-unit increase in OBS was associated with a 3% reduction in frailty risk (OR = 0.97). This protective effect remained robust after full adjustment for confounders, suggesting that a favorable oxidative balance may be an important factor in preventing frailty. Previous studies have reported that lower oxidative stress levels, as indicated by biomarkers, are significantly associated with reduced frailty risk (35). Potential mechanisms linking oxidative stress to frailty may include mitochondrial dysfunction, accelerated muscle protein degradation, increased inflammatory mediators, and accelerated cellular senescence (36). One study confirmed that the oxidative stress biomarker F2-isoprostanes was positively correlated with declines in muscle strength, providing direct evidence for this pathway (37).

The independent inverse association between CDAI and frailty risk (OR = 0.96) underscores the important role of dietary antioxidants in maintaining functional health. Notably, frailty risk was 39% lower in the highest CDAI quartile compared to the lowest, suggesting that dietary antioxidant interventions may represent a feasible strategy for frailty prevention and management. Lewis et al. reported that dietary patterns rich in polyphenols and carotenoids significantly reduced inflammatory markers in older adults (e.g., an 18% decrease in IL-6, *p* < 0.01) and improved physical performance (e.g., an 8% increase in grip strength, *p* < 0.05) (38). Dietary antioxidants may confer protection through multiple mechanisms, including free radical scavenging, inhibition of inflammatory mediator production, maintenance of mitochondrial function, and attenuation of telomere shortening (39). Cesari et al. (40) noted that the Mediterranean diet, owing to its high antioxidant content, may improve muscle mass and function and reduce frailty risk.

Our study identified non-linear associations and clear threshold effects in the relationships between OBS, CDAI, and frailty risk, which carry important clinical implications. Restricted cubic spline (RCS) analyses revealed thresholds at 9.05 for OBS and −2.39 for CDAI, suggesting the existence of biological “critical points” that may inform clinical interventions. Marron et al. similarly reported a non-linear relationship with an inflection point between antioxidant biomarkers and cognitive function, with diminishing marginal benefits beyond this point (41). The observed plateau in protective effect beyond the CDAI threshold aligns with findings by Shivappa et al. (42) regarding the Dietary Inflammatory Index, which indicated that health benefits tend to stabilize once a certain intake level is achieved. Such non-linear relationships may reflect the body’s complex regulation of oxidative-antioxidative balance. For instance, Ristow’s “mitohormesis” theory proposes that moderate oxidative stress activates endogenous antioxidant defenses, whereas excessive antioxidant intake may disrupt this adaptive mechanism (43).

The difference in the shapes of the dose–response relationships for the CDAI (linear) and OBS (non-linear with a threshold effect) can be explained by the composite nature of the OBS. While the CDAI reflects a purely dietary exposure, the OBS incorporates potent lifestyle factors such as smoking and obesity. These factors are known to contribute to systemic oxidative stress and inflammation through strong, often binary, pathways (e.g., active smoking vs. not). It is plausible that the strong threshold effect observed for the OBS is driven by these lifestyle components. This would occur when the cumulative pro-oxidant insult from both diet and lifestyle exceeds a critical capacity for homeostasis a concept aligned with the biological theory of ‘redox tipping points’ (44). Beyond this point, the body’s antioxidant defenses may be overwhelmed, leading to a sharp increase in the risk of adverse outcomes. In contrast, a purely dietary score like the CDAI reflects a more graded, subtler modulation of oxidative stress and inflammation.

It is noteworthy that although both OBS and CDAI were identified as independent protective factors, and the combination of high OBS and high CDAI was significantly associated with reduced frailty risk (OR = 0.47), the predictive model did not show significant improvement in predictive performance. The AUC of the combined model (0.577) was not significantly different from that of the OBS-alone model (0.577), and neither the Net Reclassification Improvement (NRI) nor the Integrated Discrimination Improvement (IDI) reached statistical significance. This finding is consistent with that of Buchman et al. (45), who reported that combined antioxidant supplementation (such as vitamin E and C) did not yield greater benefits compared to individual use alone. Several factors may explain these results: first, OBS and CDAI may share overlapping biological pathways, as Egea et al. (46) suggested that various antioxidant mechanisms ultimately converge on common transcription factors such as Nrf2 and NF-κB. Second, a “biological ceiling effect” may exist within the antioxidant system, whereby beyond a certain protective threshold, additional antioxidant intake provides diminishing marginal returns (47). Third, oxidative balance is a dynamic equilibrium. According to the theory of oxidative stress adaptation proposed by Salminen et al. (48), moderate oxidative stress may be necessary for maintaining cellular homeostasis.

The observed association between a higher OBS (indicating a predominance of antioxidant exposures) and a lower CDAI (indicating a less inflammatory diet) is biologically plausible, as their mechanisms are intrinsically intertwined. Although these scores were designed to capture distinct biological concepts systemic oxidative balance and dietary inflammatory potential they likely influence common pathophysiological pathways. Central to this overlap is the NF-κB signaling pathway. A pro-inflammatory diet, reflected by a higher CDAI, can directly activate NF-κB, leading to the upregulation of pro-inflammatory cytokines such as TNF-*α*, IL-1β, and IL-6 (45). Concurrently, a low OBS (indicating a pro-oxidant state) can also activate NF-κB through the excessive generation of reactive oxygen species (ROS) (49). Furthermore, many of the nutritional components emphasized in both scores, such as vitamins E and C, carotenoids, and flavonoids, possess dual anti-inflammatory and antioxidant properties. These compounds can scavenge ROS, thereby reducing oxidative stress, while also inhibiting the activation of the NF-κB and NLRP3 inflammasome pathways, consequently dampening inflammatory responses (50). This creates a bidirectional relationship where chronic inflammation promotes oxidative stress and oxidative stress perpetuates inflammation, forming a vicious cycle that contributes to cellular damage and disease progression. Therefore, the OBS and CDAI, while distinct, may act upon a shared biological axis, explaining their synergistic association with frailty in our study.

Subgroup analyses revealed that the protective effects of both OBS and CDAI remained consistent across diverse populations, reinforcing the robustness and generalizability of the findings. Notably, the combined protective effect was more pronounced among adults aged ≥65 years (OR = 0.45) and those with diabetes (OR = 0.41). This supports the observation reported by Neymotin and Sen (51) that high-risk populations may derive greater benefit from antioxidant interventions. This may be attributed to higher baseline oxidative stress and more rapid depletion of antioxidant reserves in older adults and individuals with metabolic abnormalities, which could enhance their responsiveness to exogenous antioxidants (52). Similarly, El Assar et al. (53) highlighted that age-related endothelial dysfunction is closely associated with elevated oxidative stress, and antioxidant strategies may be particularly effective in older populations.

A notable finding of our study was the significant effect modification by sex, with the inverse associations between OBS, CDAI, and frailty being substantially stronger in women than in men. Several non-mutually exclusive mechanisms could explain this disparity. Biologically, women may exhibit a different oxidative stress response and a higher susceptibility to oxidative damage compared to men, potentially due to differences in hormone profiles (e.g., the decline in estrogen during menopause), body composition, and mitochondrial function. Consequently, dietary antioxidants may have a more pronounced impact on mitigating this underlying vulnerability. From a behavioral perspective, women generally report higher diet quality and greater adherence to healthy dietary patterns than men, which might lead to a more effective translation of antioxidant and anti-inflammatory nutrient intake into biological benefit. Furthermore, sex differences in body composition, fat distribution, and frailty criteria prevalence (e.g., higher rates of sarcopenia in men versus higher rates of exhaustion and low activity in women) could also contribute to the differential associations observed. This finding underscores the importance of considering sex as a critical factor in developing nutritional strategies for frailty prevention, suggesting that such interventions might need to be tailored to be most effective.

This study has several notable strengths. First, the identification of specific threshold values for OBS and CDAI offers quantitative benchmarks for developing personalized intervention strategies, allowing clinicians to tailor precise nutritional and lifestyle recommendations for patients with early-stage CKM. Second, the detection of non-linear relationships suggests that interventions should initially focus on individuals below these thresholds, as they are likely to experience the greatest health benefits. Third, the absence of a significant additive effect implies that in resource-limited settings, simultaneous improvement of both OBS and CDAI may not be necessary; instead, interventions can be prioritized based on individual patient profiles. Furthermore, this study provides new risk stratification tools for early-stage CKM patients, enabling earlier identification of high-risk individuals and facilitating targeted interventions.

There are, however, several limitations to this study. Firstly, the cross-sectional nature of the NHANES data limits our ability to infer causality, as we cannot determine whether the observed dietary patterns preceded the development of frailty. Therefore, our results suggest a significant association but not a causal relationship. Future prospective studies are needed to confirm these findings and explore the potential causative role of an anti-inflammatory diet. Secondly, despite adjustments for numerous confounders, residual confounding may still exist, such as unmeasured genetic or environmental factors. Thirdly, the calculations of OBS and CDAI were based on self-reported dietary and lifestyle data, which may be subject to recall bias; future studies should consider incorporating objective biomarkers to assess oxidative balance. Fourthly, our study is subject to the limitations inherent in analyzing composite nutritional indices. While the OBS and CDAI provide holistic measures of dietary patterns, the components within these scores are interrelated. Although statistical checks (VIF < 5) indicated that multicollinearity did not critically inflate variance in our final models, the correlation between constituents remains an inherent feature of the exposure. This complicates the mechanistic interpretation of which specific dietary factors or biological pathways are most responsible for the observed associations with frailty. The findings should therefore be interpreted as reflecting the effect of the overall dietary and antioxidant pattern rather than its isolated components. Fifthly, although the combined model demonstrated a statistically significant improvement in discriminative ability over the CDAI-only model (ΔAUC = 0.012, *p* = 0.0114), the absolute magnitude of this improvement was modest. This warrants caution in interpreting its immediate clinical relevance and utility. The incremental value of adding these biomarkers for risk stratification in routine clinical practice should be further validated in prospective studies and assessed through impact analyses that measure effects on clinical decision-making and patient outcomes. Sixthly, our study used the CDAI, which is based solely on dietary intake and does not account for the use of vitamin or mineral supplements. This is an inherent limitation of the CDAI construct, which aims to isolate the inflammatory potential of the diet itself. Consequently, our analysis may underestimate the total antioxidant and anti-inflammatory exposure for individuals who regularly use supplements, potentially leading to an underestimation of the true association between anti-inflammatory intake and frailty. Future studies would benefit from creating an expanded index that integrates comprehensive data on supplement usage, such as that available in NHANES, to more accurately quantify total exposure and further elucidate the relationship between combined dietary and supplemental anti-inflammatory intake and health outcomes. Finally, we acknowledge that the relatively low AUC values across all models indicate limited discriminatory accuracy of OBS and CDAI as standalone predictive tools for frailty risk at the individual level. This is not entirely unexpected, given that frailty is a complex multifactorial syndrome unlikely to be fully captured by any single or pair of biomarkers. However, the consistent and graded associations observed in this study remain valuable from a public health perspective, as they suggest that dietary and oxidative stress factors may contribute to frailty risk at the population level, even if their predictive power for individual risk stratification is modest.

In conclusion, our cross-sectional analysis of a nationally representative sample suggests that higher dietary antioxidant and anti-inflammatory potential, as measured by OBS and CDAI, may be associated with a lower prevalence of frailty among older U. S. adults. These observed associations were independent of major sociodemographic and health-related confounders and exhibited dose–response relationships. However, given the observational design of our study, we cannot rule out residual confounding or establish causality. Furthermore, the modest predictive accuracy of our models indicates that these indices are not, by themselves, sufficient for individual-level risk prediction. Nonetheless, our findings contribute to the growing body of evidence linking diet and oxidative stress to healthy aging and suggest that promoting antioxidant-rich and anti-inflammatory diets represents a promising, modifiable target for public health strategies aimed at reducing frailty risk at a population level. Future prospective longitudinal studies and randomized controlled trials are warranted to confirm these associations and elucidate the underlying mechanisms.

## Data Availability

The original contributions presented in the study are included in the article/Supplementary material, further inquiries can be directed to the corresponding author.

## Glossary

NHANES: National Health and Nutrition Examination Survey
PIR: Poverty income ratio
BMI: Body mass index
HbA1c: Glycosylated hemoglobin
UA: Uric acid
BUN: Blood urea nitrogen
TG: Triglyceride
TC: Total cholesterol
HDL: High density lipoprotein
LDL: Low density lipoprotein
eGFR: Estimated glomerular filtration rate
FPG: Fasting plasma glucose
OBS: Oxidative balance score
CDAI: Composite dietary antioxidant index
CKM: Cardiovascular-kidney-metabolic
NRI: Net reclassification improvement
IDI: Integrated discrimination improvement
RCS: Restricted cubic spline
ROC: Receiver operating characteristic curve
AUC: Area under curve
