# Supplementary material for: Non-linear associations and threshold effects of oxidative balance score and composite dietary antioxidant index on frailty risk in patients with cardiovascular-kidney-metabolic syndrome
Source: Front Nutr. 2025 Nov 3;12:1673736. doi: 10.3389/fnut.2025.1673736 (PMC12620448; doi:10.3389/fnut.2025.1673736)
Supplement: Supplementary file 1 [file Table_1.DOC]

**Supplementary Table 1. Definition of CKM**

| CKM stages | Definition |
| --- | --- |
| CKM stage 0 | Participants with a normal Body Mass Index (BMI) (<23 kg/m² for those of Asian ethnicity and <25 kg/m² for individuals from other racial and ethnic backgrounds), and a normal waist circumference (<80 cm for Asian women and <90 cm for Asian men, or <88 cm for women and <102 cm for men in all other racial and ethnic groups) who did not fulfill the criteria for the other stages. |
| CKM stage 1 | Elevated Body Mass Index (BMI) (≥23 kg/m² for individuals of Asian descent and >25 kg/m² for all other racial and ethnic groups), increased waist circumference (≥80 cm for Asian women and ≥90 cm for Asian men, or ≥88 cm for women and ≥102 cm for men in other racial and ethnic categories), or prediabetes. Prediabetes is defined as a glycated hemoglobin (HbA1c) level of 5.7% to <6.5% or a fasting blood glucose level between 100 mg/dL and <126 mg/dL. |
| CKM stage 2 | Metabolic risk factors or moderate-to-high-risk chronic kidney disease (CKD) as defined by the Kidney Disease: Improving Global Outcomes (KDIGO) criteria, in accordance with AHA recommendations. The qualifying metabolic risk factors encompassed: elevated fasting serum triglycerides (≥135 mg/dL) ; hypertension; diabetes; metabolic syndrome, characterized by the presence of at least three of the following:  increased waist circumference; reduced high-density lipoprotein (HDL) cholesterol levels (<40 mg/dL for men, <50 mg/dL for women); fasting serum triglycerides ≥150 mg/dL; elevated blood pressure (systolic ≥130 mmHg, diastolic ≥80 mmHg, and/or use of antihypertensive medication); prediabetes. |
| CKM stage 3 | Presence of very-high-risk KDIGO CKD stages or a high-estimated 10-year cardiovascular disease (CVD) risk. The 10-year CVD risk was assessed using the AHA PREVENT equations for predicting cardiovascular events. A 10-year CVD risk of 20% or greater was classified as high risk. |
| CKM stage 4 | Reported history of established cardiovascular conditions, including coronary heart disease, angina, myocardial infarction, heart failure, and cerebrovascular accident. |

**Supplementary Table 2. Items in the 49-item Frailty Index and corresponding detailed scoring criteria.**

| **Defects** | **Scoring** |
| --- | --- |
| **Cognition** | |
| 1. Experience confusion/memory problems | Yes = 1  No = 0 |
| **Dependence** | |
| 2. Managing money | Difficulty = 1  No Difficulty = 0 |
| 3. Stooping, crouching, kneeling |
| 4. Lifting or carrying |
| 5. House chore |
| 6. Preparing meals |
| 7. Standing up from armless chair |
| 8. Getting in and out of bed difficulty |
| 9. Using fork, knife, drinking from cup |
| 10. Dressing yourself |
| 11. Standing for long periods difficulty |
| 12. Grasp/holding small objects |
| 13. Attending social events |
| 14. Push or pull large objects |
| 15. Walking for a quarter mile difficulty |
| 16. Walking up 10 steps difficulty |
| **Depressive Symptoms** | |
| 17. Have little interest in doing things | Nearly every day = 1  More than half the days = 0.66  Several days = 0.33  Not at all = 0 |
| 18. Feeling down, depressed, or hopeless |
| 19. Trouble sleeping or sleeping too much |
| 20. Feeling tired or having little energy |
| 21. Poor appetite or overeating |
| 22. Feeling bad about yourself |
| 23. Trouble concentrating on things |
| **Comorbidities** | |
| 24. Arthritis | Yes = 1  Suspect = 0.5  No = 0 |
| 25. Thyroid problems |
| 26. Chronic bronchitis |
| 27. Cancer |
| 28. Congestive heart failure |
| 29. Coronary heart disease |
| 30. Angina |
| 31. Heart attack |
| 32. Stroke |
| 33. Blood pressure |
| 34. Diabetes |
| 35. Weak/failing kidneys |
| 36. Urinary Leakage |
| **Hospital Utilization and Access to Care** | |
| 37. Self-rated health | Fair or poor = 1  Excellent, very good, or good = 0 |
| 38. Health now compared with 1 year ago | Worse = 1  About the same, Better = 0 |
| 39. Overnight hospital patient in past year | Yes = 1  No = 0 |
| 40. Frequency of healthcare use during the past year | None = 0, 1 to 5 = 0.5, More than 5 = 1 |
| 41. Number of prescribed medications | None = 0, 1 to 4 = 0.5, ≥5 = 1 |
| **Physical Performance and Anthropometry** | |
| 42. Body mass index | <18.5 or ≥30 = 1  25 to 30 = 0.5  18.5 to 25 = 0 |
| 43. Handgrip strength | Male:  For BMI ≤ 24, GS ≤ 29 = 1;  For BMI 24 to 28, GS ≤ 30 = 1;  For BMI >28, GS ≤ 32 = 1.  Female:  For BMI ≤23, GS ≤17 = 1;  For BMI 23 to 26, GS ≤17.3 = 1;  For BMI 26 to 29, GS ≤ 18 = 1;  For BMI>29, GS ≤ 21 = 1. |
| **Laboratory Values** | |
| 44. Glycohemoglobin (%) | 0% to 5.7% = 0, >5.7% = 1 |
| 45. Red blood cell count **(million cells/ml)** | Male: 4.7 to 6.1 = 0, Other = 1  Female: 4.2 to 5.4 = 0, Other = 1 |
| 46. Hemoglobin (g/dL) | Male: 13.5 to 18 = 0, Other = 1  Female: 12 to 16 = 0, Other = 1 |
| 47. Red cell distribution width (%) | 11.6 to 14.6 = 0, Other = 1 |
| 48. Lymphocyte percent (%) | 20 to 40 = 0, Other = 1 |
| 49. Segmented neutrophils percent (%) | 40 to 80 = 0, Other = 1 |

BMI, Body mass index; GS, grip strength.

**Supplementary Table 3.Collinearity diagnosis**

| **Variables** | **Β-Value** | **VIF** |
| --- | --- | --- |
| **Age** | -0.011 | 2.589 |
| **Sex** | -0.116 | 1.158 |
| **Race** | -0.014 | 1.056 |
| **Hypertension** | 0.108 | 1.837 |
| **Diabetes** | 0.011 | 1.197 |
| **BMI** | 0.085 | 1.248 |
| **HbA1c** | 0.059 | 1.577 |
| **TG** | 0.023 | 1.296 |
| **HDL** | -0.011 | 1.502 |
| **Antidiabetic** | 0.127 | 1.655 |
| **Antihypertension** | 0.088 | 2.180 |
| **Antihyperlipidemic** | 0.026 | 1.440 |
| **eGFR** | -0.065 | 2.097 |
| **OBS** | -0.060 | 1.326 |
| **CDAI** | -0.020 | 1.307 |

**Supplementary Table 4.Sensitivity analysis to exclude the association between OBS, CDAI and risk of frailty in patients with early CKM after excluding patients with CKM Stage 0**

| **Variables** | **Model 1** | | **Model 2** | | **Model 3** | |
| --- | --- | --- | --- | --- | --- | --- |
|  | **OR(95%CI)** | **P** | **OR(95%CI)** | **P** | **OR(95%CI)** | **P** |
| **OBS** | 0.97(0.95,0.99) | <0.001 | 0.97(0.96,0.98) | <0.001 | 0.97(0.96,0.98) | <0.001 |
| **OBSQ** | | | | | | |
| **Q1** | Ref | Ref | Ref | Ref | Ref | Ref |
| **Q2** | 0.79(0.64,0.99) | 0.040 | 0.70(0.56,0.89) | 0.003 | 0.68(0.53,0.88) | 0.004 |
| **Q3** | 0.65(0.52,0.82) | <0.001 | 0.58(0.46,73) | <0.001 | 0.58(0.45,0.74) | <0.001 |
| **Q4** | 0.50(0.40,0.63) | <0.001 | 0.45(0.35,0.57) | <0.001 | 0.50(0.39,0.65) | <0.001 |
| **P for trend** | <0.001 | | <0.001 | | <0.001 | |
|  | | | | | | |
| **CDAI** | 0.95(0.93,0.97) | <0.001 | 0.96(0.94,0.98) | <0.001 | 0.96(0.94,0.98) | <0.001 |
| **CDAIQ** | | | | | | |
| **Q1** | Ref | Ref | Ref | Ref | Ref | Ref |
| **Q2** | 0.76(0.63,0.93) | 0.010 | 0.76(0.62,0.93) | 0.010 | 0.74(0.59,0.93) | 0.010 |
| **Q3** | 0.62(0.51,0.76) | <0.001 | 0.65(0.53,0.79) | <0.001 | 0.63(0.51,0.78) | <0.001 |
| **Q4** | 0.60(0.49,0.73) | <0.001 | 0.65(0.53,0.79) | <0.001 | 0.63(0.52,0.77) | <0.001 |
| **P for trend** | <0.001 | | <0.001 | | <0.001 | |

**OR: Odds ratio, CI: confidence interval, Ref: reference**

**Model 1: No adjustments made;**

**Model 2: Adjusted for Age, Sex, Race;**

**Model 3:Adjusted for Age,Sex,Race,Hypertension,Diabetes,Antidiabetic,Antihypertension,Antihyperlipidemic,BMI,eGFR,HbA1c,TG,HDL**
